# Supplementary material for: Chip-Based Spectrofluorimetric Determination of Iodine in a Multi-Syringe Flow Platform with and without In-Line Digestion—Application to Salt, Pharmaceuticals, and Algae Samples
Source: Molecules. 2022 Feb 16;27(4):1325. doi: 10.3390/molecules27041325 (PMC8877039; doi:10.3390/molecules27041325)
Supplement: Supplementary file 1 [file molecules-27-01325-s001.zip › molecules-1600964-supplementary.pdf]

Supplementary Material

Chip-Based Spectrofluorimetric Determination of Iodine in a Multi-Syringe Flow Platform with and without In-Line Digestion—Application to Salt, Pharmaceuticals, and Algae Samples

Joana L. A. Miranda [1], Raquel B. R. Mesquita [1], Edwin Palacio [2], José M. Estela [2], Víctor Cerdà [3] and António O. S. S. Rangel [1,*]

[1] CBQF—Centro de Biotecnologia e Química Fina—Laboratório Associado, Escola Superior de Biotecnologia, Universidade Católica Portuguesa, Rua Diogo Botelho 1327, 4169-005 Porto, Portugal; jmiranda@ucp.pt (J.L.A.M.); rmesquita@ucp.pt (R.B.R.M.)

[2] Department of Chemistry, University of the Balearic Islands, E-07122, Palma de Mallorca, Spain; edwin.palacio@uib.es (E.P.); josemanuel.estela@uib.es (J.M.E.)

[3] Sciware Systems S.L. Bunyola, 07193. Spain, victorcerdamartin@gmail.com

* Correspondence: arangel@ucp.pt

Table S1. Some analytical characteristics of flow methods for the determination of iodine in different sample matrices.

Flow System	Method Characteristics	Detection	Matrix	Application range	LOD	Reference
SFA	Sandell-Kolthoff reaction	Spectrophotometry	urine	50–400 μg L^{-1}	-	[1]
FIA	Chemiluminescence reaction	Spectrophotometry	urine	0–5000 μg L^{-1} 10–40 mg L^{-1}	10 μg L^{-1}	[2]
FIA	Chemiluminescence reaction	Spectrophotometry	salt	126.9–1522.8 μg L^{-1}	12.7 μg L^{-1}	[3]
FIA	-	Potentiometry	urine and salt	0.317–126.9 (iodide) mg L^{-1} 0.444–253.8 (iodide + iodate) mg L^{-1}	176 (iodide) μg L^{-1} 225 (iodide+iodate) μg L^{-1}	[4]
FIA	Sandell-Kolthoff reaction	Spectrophotometry	urine	12.7–380.7 μg L^{-1}	12.7 μg L^{-1}	[5]
FIA	Sandell-Kolthoff reaction	Spectrophotometry	urine samples	4.95–40.0 μg L^{-1} 40.0–1001 μg L^{-1}	4.95 μg L^{-1}	[6]
FIA	Chemiluminescence reaction	Spectrophotometry	salt and pharmaceutical	1.3–63.5 μg L^{-1}	0.13 μg L^{-1}	[7]
Continuous and stopped FIA	Sandell-Kolthoff reaction	Spectrophotometry	urine samples	20–200 μg L^{-1} continuous 50–200 μg L^{-1} stopped	2.3 μg L^{-1} continuous 3 μg L^{-1} stopped	[8]
FIA	Liquid-liquid extraction	Spectrophotometry	biodiesel	13–135g I_2/ 100g	5 g I_2/ 100g	[9]
FIA	Polyvinyl alcohol reagent	Spectrophotometry	seawater and salt	1–12.7 μg m L^{-1}	60 ng m L^{-1}	[10]
FIA	-	Spectrophotometry	seawater and salt	0.51–4.06 μg m L^{-1}	40 ng m L^{-1}	[11]
FIA	Oxidation with tartaric acid	Potentiometry	-	-	25.4 μg L^{-1}	[12]
FIA	Gas diffusion unit	Potentiometry	-	up till 1269 μg L^{-1}	25.4 μg L^{-1}	[13]
FIA	Colorimetric reagent	Spectrophotometry	water recovery system samples	0.01–4 mg L^{-1}	52 μg L^{-1}	[14]
FIA	Sandell-Kolthoff reaction	Spectrophotometry	bottled drinking water	50–1000 μg L^{-1}	9.30 μg L^{-1}	[15]
FIA	-	Amperometry	iodized table salt	0–25 mg L^{-1}	0.5 mg L^{-1}	[16]
FIA	Sandell-Kolthoff reaction	Spectrophotometry	seawater and pharmaceutical preparation	4.3–70 μg L^{-1}	0.47 μg L^{-1}	[17]
FIA	Catalytic reaction	Spectrophotometry	milk	0–100 μg L^{-1}	0.99 μg L^{-1}	[18]
FIA	Iron(II)-tris bathophenanthroline iodide ion pair complex	Potentiometry	pharmaceutical preparations	1.3–1200 mg L^{-1}	0.5 mg L^{-1}	[19]
FIA	Iodine– starch reaction (gas diffusion unit)	Spectrophotometry	pharmaceutical	6000–10000 mg L^{-1}	1 mg L^{-1}	[20]
FIA	Sandell-Kolthoff reaction	Spectrophotometry	egg	-	0.58 μg g^{-1} egg sample	[21]
FIA	Hanus reagent	Amperometry	vegetable oils	5–12 mg L^{-1}	6.3 mg L^{-1}	[16]
FIA	Parallel flow	Spectrophotometry	olive oil	90–125 g L^{-1}	38 g L^{-1}	[22]
FIA	Chemiluminescence reaction with gas-diffudion unit	Spectrophotometry	pharmaceutical	0.1–1.0 mg L^{-1}	0.1 mg L^{-1}	[23]
FIA	Sandell-Kolthoff reaction	Spectrophotometry	thyroid gland	5–200 μg L^{-1}	1.6 μg L^{-1}	[24]
FIA	blue $I_3(-)$ - starch complex	Spectrophotometry	iodized salt	0.63–5.1 mg L^{-1}	2 mg kg^{-1}	[25]
FIA	gas-diffusion cell	Spectrophotometry	marine pore water	0–400 mg L^{-1}	0.2 mg L^{-1}	[26]
FIA	Catalytic reaction	Spectrophotometry	sea water	0.75–150 μg L^{-1}	-	[27]
FIA	Iodate-acid reaction	Spectrophotometry	charcoals	0–317 mg L^{-1}	7.6 mg L^{-1}	[28]
FIA	Chemiluminescence reaction	Spectrophotometry	-	0.05–10 μg L^{-1}	0.05 μg L^{-1}	[29]
FIA	Catalytic reaction	Spectrophotometry	water	0.1–2 μg L^{-1}	0.1 μg L^{-1}	[30]

Flow System	Method Characteristics	Detection	Matrix	Application range	LOD	Reference
FIA	-	Potentiometry	pharmaceutical preparations	0.1269–12690 mg L^{-1} (iodide) 1.269–126.9 mg L^{-1} (iodine)	0.6345 mg L^{-1} (iodide) 1.269 mg L^{-1} (iodine)	[31]
FIA	Chemiluminescence reaction	Spectrophotometry	multivitamin tablets	1.0–10.0 mg L^{-1}	0.5 mg L^{-1}	[32]
FIA	Catalytic reaction	Spectrophotometry	stream sediments	0.060–0.150 mg L^{-1}	0.4 mg L^{-1}	[33]
FIA	-	Potentiometry	iodized table salts	3.2–95.2 mg L^{-1}	0.3 mg L^{-1}	[34]
FIA	-	Amperometry	water, serum, salt	0.0635–12,7 mg L^{-1}	0.63 μg L^{-1}	[35]
FIA	Chemiluminescence reaction	Spectrophotometry	salt	0.13–1.5 mg L^{-1}	12.7 μg L^{-1}	[3]
MSFIA	Spectrofluorimetric method - reaction with Alizarin Navy Blue Spectrophotometric method - reaction with iodide in acidic media	Spectrophotometry and Spectrofluorimetry	artificial fresh water	periodate 1.3–28 mg L^{-1} iodate 1.3–25 mg L^{-1}	periodate 0.44 mg L^{-1} iodate 0.13 mg L^{-1}	[36]
MSFIA	Sandell-Kolthoff reaction	Spectrofluorimetry	seawater	1–100 μg L^{-1}	0.3 μg L^{-1}	[37]
MFFIA	Sandell-Kolthoff reaction	Spectrofluorimetry	drinking water	50–400 μg L^{-1}	7.7 μg L^{-1}	[38]
SIA	Catalytic reaction	Spectrophotometry	tablets	0.1–6.0 μg L^{-1}	0.05 μg L^{-1}	[39]
SIA	-	Potentiometry	bathing waters and seaweed extracts	634.5–63450 μg L^{-1}	17.8 μg L^{-1} (iodide) 2.54 μg L^{-1} (iodate)	[40]

SFA - Segmented flow analysis
FIA - Flow injection analysis
MSFIA – Multi-syringe flow injection system
MFFIA – Microfluidic flow injection system
SIA –Sequential injection analysis

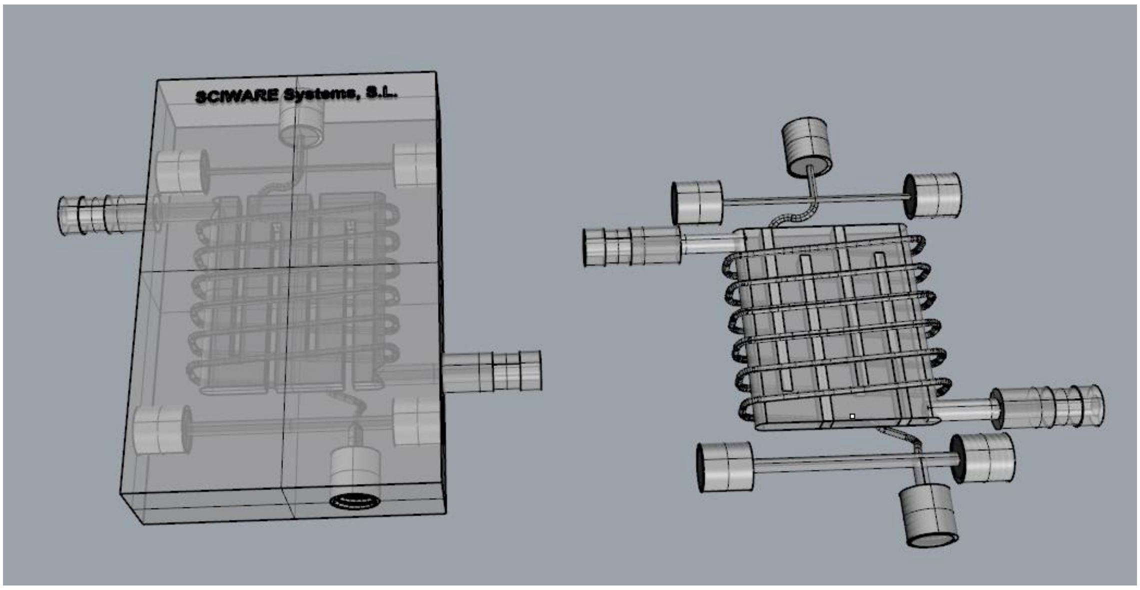

Figure S1. Structure scheme of the Chip construction with the Rhinoceros software.

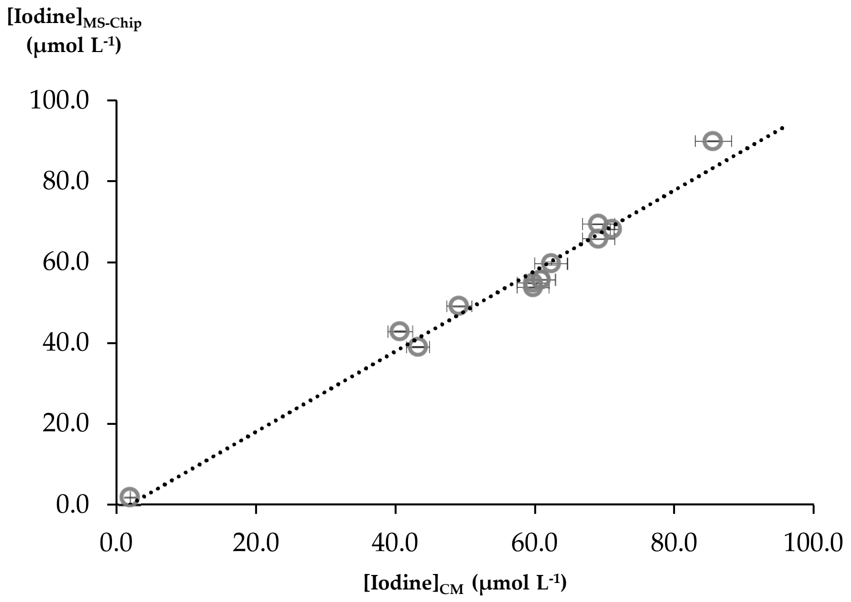

Figure S2. Comparison of the results obtained with the proposed MS-Chip method $[Iodine]_{MS-Chip}$ and with the comparison methods $[Iodine]_{CM}$ (potentiometric detection method for iodide and iodometric titration method for iodate quantification) for the analysis of marine salt samples.

References

1. Garry, P.J.; Lashley, D.W.; Owen, G.M. Automated Measurement of Urinary Iodine. *Clin. Chem.* **1973**, *19*, 950–953; doi: 10.1093/clinchem/19.9.950.
2. Burguera, J.L.; Brunetto, M.R.; Contreras, Y.; Burguera, M.; Gallignani, M.; Carrero, P. Head-space flow injection for the on-line determination of iodide in urine samples with chemiluminescence detection. *Talanta* **1996**, *43*, 839–850, doi:10.1016/0039-9140(95)01722-4.
3. Yaqoob, M.; Atiq-Ur-Rehman; Waseem, A.; Nabi, A. Determination of iodide using flow injection with acidic potassium permanganate chemiluminescence detection. *Luminescence* **2006**, *21*, 221–225, doi:10.1002/bio.910.
4. Machado, A.; Mesquita, R.B.R.; Oliveira, S.; Bordalo, A.A. Development of a robust, fast screening method for the potentiometric determination of iodide in urine and salt samples. *Talanta* **2017**, *167*, 688–694, doi:10.1016/j.talanta.2017.03.017.
5. Tsuda, K.; Namba, H.; Nomura, T.; Yokoyama, N.; Yamashita, S.; Izumi, M.; Nagataki, S. Automated measurement of urinary iodine with use of ultraviolet irradiation. *Clin. Chem.* **1995**, *41*, 581–585, doi:10.1093/clinchem/41.4.581.
6. Yaping, Z.; Dongxing, Y.; Jixiang, C.; Tianshiu, L.; Huiqin, C. Spectrophotometric determination of urinary iodine by flow-injection analysis with on-line catalytic digestion. *Clin. Chem.* **1996**, *42*, 2021–2027, doi:10.1093/clinchem/42.12.2021.
7. Waseem, A.; Yaqoob, M.; Nabi, A. Flow-injection method for the determination of iodide/iodine using Ru(bpy)33+-NADH chemiluminescence detection. *Luminescence* **2008**, *23*, 316–320, doi:10.1002/BIO.1037.
8. Nacapricha, D.; Muangkaew, S.; Ratanawimarnwong, N.; Shiowatana, J.; Grudpan, K. Continuous and stopped flow injection for catalytic determination of total iodine in urine. *Analyst* **2001**, *126*, 121–126, doi:10.1039/b007131p.
9. Pereira, A.C.; Rocha, F.R.P. Liquid-liquid microextraction in a multicommuted flow system for direct spectrophotometric determination of iodine value in biodiesel. *Anal. Chim. Acta* **2014**, *829*, 28–32, doi:10.1016/J.ACA.2014.04.049.
10. Kuznetsov, V. V.; Ermolenko, Y. V.; Seffar, L. Flow-injection determination of elemental iodine by polyvinyl alcohol. *J. Anal. Chem.* **2004**, *59*, 688–693, doi:10.1023/B:JANC.0000035285.81722.2F.
11. Kuznetsov, V. V.; Ermolenko, Y. V.; Seffar, L. Amylose and amylopectin as reagents for the flow-injection determination of elemental iodine. *J. Anal. Chem. 2007 625* **2007**, *62*, 479–485, doi:10.1134/S1061934807050164.
12. Trojánek, A.; Papoff, P. Pneumatoamperometric flow-injection determination of iodide. *Anal. Chim. Acta* **1991**, *247*, 73–77, doi:10.1016/S0003-2670(00)83054-8.
13. Motomizu, S.; Yoden, T. Porous membrane permeation of halogens and its application to the determination of halide ions and residual chlorine by flow-injection analysis. *Anal. Chim. Acta* **1992**, *261*, 461–469, doi:10.1016/0003-2670(92)80227-X.
14. Williamson, J.P.; Emmert, G.L. A flow injection analysis system for monitoring silver (I) ion and iodine residuals in recycled water from recovery systems used for spaceflight. *Anal. Chim. Acta* **2013**, *792*, 72–78, doi:10.1016/J.ACA.2013.07.011.
15. Choengchan, N.; Lukkanakul, K.; Ratanawimarnwong, N.; Waiyawat, W.; Wilairat, P.; Nacapricha, D. Use of pseudo-first order kinetics in flow injection for determination of trace inorganic iodine. *Anal. Chim. Acta* **2003**, *499*, 115–122, doi:10.1016/J.ACA.2003.08.062.
16. Jakmunee, J.; Grudpan, K. Flow injection amperometry for the determination of iodate in iodized table salt. *Anal. Chim. Acta* **2001**, *438*, 299–304, doi:10.1016/S0003-2670(01)00798-X.

17. Abouhiat, F.Z.; Henriquez, C.; Horstkotte, B.; El Yousfi, F.; Cerda, V. A miniaturized analyzer for the catalytic determination of iodide in seawater and pharmaceutical samples. *Talanta* **2013**, *108*, 92–102, doi:10.1016/j.talanta.2013.02.072.
18. De Nogueira, A.R.A.; Mockiuti, F.; De Souza, G.B.; Primavesi, O. Flow Injection Spectrophotometric Catalytic Determination of Iodine in Milk. *Anal. Sci.* **1998**, *14*, 559–564, doi:10.2116/ANALSCI.14.559.
19. Hassan, S.S.M.; Marzouk, S.A.M. Sequential flow-injection potentiometric determination of iodide and iodine in povidone iodine pharmaceuticals. *Electroanalysis* **1993**, *5*, 855–861, doi:10.1002/ELAN.1140050921.
20. Nacapricha, D.; Uraisin, K.; Ratanawimarnwong, N.; Grudpan, K. Simple and selective method for determination of iodide in pharmaceutical products by flow injection analysis using the iodine-starch reaction. *Anal. Bioanal. Chem.* **2004**, *378*, 816–821, doi:10.1007/S00216-003-2370-7/FIGURES/3.
21. Srivorakul, T.; Varanusupakul, P.; Alahmad, W. Development of a Sample Treatment Method for a Flow Injection Determination of Iodine in Eggs: A Comparison Study. *Anal. Sci.* **2020**, *36*, 491–495, doi:10.2116/ANALSCI.19N028.
22. Thomaidis, N.S.; Georgiou, C.A. Direct parallel flow injection multichannel spectrophotometric determination of olive oil iodine value. *Anal. Chim. Acta* **2000**, *405*, 239–245, doi:10.1016/S0003-2670(99)00711-4.
23. Ratanawimarnwong, N.; Amornthammarong, N.; Choengchan, N.; Chaisuwan, P.; Amatatongchai, M.; Wilairat, P.; McKelvie, I.D.; Nacapricha, D. Determination of iodide by detection of iodine using gas-diffusion flow injection and chemiluminescence. *Talanta* **2005**, *65*, 756–761, doi:10.1016/J.TALANTA.2004.08.002.
24. Zabala, J.; Carrión, N.; Murillo, M.; Quintana, M.; Chirinos, J.; Seijas, N.; Duarte, L.; Brätter, P. Determination of normal human intrathyroidal iodine in Caracas population. *J. Trace Elem. Med. Biol.* **2009**, *23*, 9–14, doi:10.1016/J.JTEMB.2008.11.002.
25. Choengchan, N.; Uraisin, K.; Choden, K.; Veerasai, W.; Grudpan, K.; Nacapricha, D. Simple flow injection system for colorimetric determination of iodate in iodized salt. *Talanta* **2002**, *58*, 1195–1201, doi:10.1016/S0039-9140(02)00438-1.
26. Håkedal, J.T.; Egeberg, P.K. Determination of Iodide in Brines by Membrane Permeation Flow Injection Analysis†. *Analyst* **1997**, *122*, 1235–1238, doi:10.1039/A704014H.
27. Oguma, K.; Kitada, K.; Kuroda, R. Microchemical determination of lodate and iodide in sea waters by flow injection analysis. *Microchim. Acta 1993 1101* **1993**, *110*, 71–77, doi:10.1007/BF01243987.
28. Monks, C.D.; Nacapricha, D.; Taylor, C.G. Determination of iodide ion in impregnated charcoals by flow injection. *Analyst* **1993**, *118*, 623–626, doi:10.1039/AN9931800623.
29. Fujiwara, T.; Mohammadzai, I.U.; Kojima, M.; Kumamaru, T. An improved method for the flow-injection determination of iodine using the luminol chemiluminescence reaction in a reversed micellar medium of cetyltrimethylammonium chloride in 1-hexanol-cyclohexane. *Anal. Sci.* **2006**, *22*, 67–71, doi:10.2116/ANALSCI.22.67.
30. Yonehara, N.; Kozono, S.; Sakamoto, H. Flow Injection-Spectrophotometric Determination of Trace Amounts of Iodide by Its Catalytic Effect on the 4, 4′-Bis(dimethylamino)-diphenylmethane- Chioramine T Reaction. *Anal. Sci.* **1991**, *7*, 229–234, doi:10.2116/ANALSCI.7.229.
31. Davey, D.E.; Mulcahy, D.E.; O'Connell, G.R. Potentiometric flow-injection determination of iodide and iodine. *Talanta* **1990**, *37*, 313–316, doi:10.1016/0039-9140(90)80059-O.
32. Nacapricha, D.; Sangkarn, P.; Karuwan, C.; Mantim, T.; Waiyawat, W.; Wilairat, P.; Cardwell, T.; McKelvie, I.D.; Ratanawimarnwong, N. Pervaporation-flow injection with chemiluminescence detection for determination of iodide in multivitamin tablets. *Talanta* **2007**, *72*, 626–633, doi:10.1016/J.TALANTA.2006.11.033.
33. Liu, G.; Li, J.; Zhao, X. Ion Exchange-Flow Injection Spectrometric Simultaneous Determination of Traces of Bromide and Iodide in some Chinese Standard Samples. *Geostand. Newsl.* **1995**, *19*, 215–220, doi:10.1111/J.1751-908X.1995.TB00159.X.
34. Nellaiappan, S.; Kumar, A.S. Selective flow injection analysis of iodate in iodized table salts by riboflavin immobilized multiwalled carbon nanotubes chemically modified electrode. *Electrochim. Acta* **2013**, *109*, 59–66, doi:10.1016/J.ELECTACTA.2013.07.076.
35. Nikolic, S.D.; Mutic, J.J.; Lolic, A.D.; Manojlovic, D.D. Sensitive flow-injection amperometric detection of iodide using Mn3+ and As3+. *Anal. Sci.* **2005**, *21*, 525–529, doi:10.2116/analsci.21.525.
36. Ensafi, A.A.; Dehaghi, G.B. Flow-injection simultaneous determination of iodate and periodate by spectrophotometric and spectrofluorometric detection. *Anal. Sci.* **2000**, *16*, 61–64, doi:10.2116/ANALSCI.16.61.
37. Frizzarin, R.M.; Aguado, E.; Portugal, L.A.; Moreno, D.; Estela, J.M.; Rocha, F.R.P.; Cerda, V. A portable multi-syringe flow system for spectrofluorimetric determination of iodide in seawater. *Talanta* **2015**, *144*, 1155–1162, doi:10.1016/j.talanta.2015.07.069.
38. Inpota, P.; Strzelak, K.; Koncki, R.; Sripumkhai, W.; Jeamsaksiri, W.; Ratanawimarnwong, N.; Wilairat, P.; Choengchan, N.; Chantiwas, R.; Nacapricha, D. Microfluidic Analysis with Front-Face Fluorometric Detection for the Determination of Total Inorganic Iodine in Drinking Water. *Anal. Sci.* **2018**, *34*, 161–167, doi:10.2116/analsci.34.161.
39. Tesfaldet, Z.O.; Van Staden, J.F.; Stefan, R.I. Sequential injection spectrophotometric determination of trace amounts of iodide by its catalytic effect on the 4,4′-methylenebis(N,N- dimethylaniline)-chloramine-T reaction. *Talanta* **2004**, *64*, 1213–1219, doi:10.1016/J.TALANTA.2004.04.038.
40. Santos, I.C.; Mesquita, R.B.R.; Bordalo, A.A.; Rangel, A. Iodine speciation in coastal and inland bathing waters and seaweeds extracts using a sequential injection standard addition flow-batch method. *Talanta* **2015**, *133*, 7–14, doi:10.1016/j.talanta.2014.01.025.
